# *BRCA2* mutations should be screened early and routinely as markers of poor prognosis: evidence from 8,988 patients with prostate cancer

**DOI:** 10.18632/oncotarget.16712

**Published:** 2017-03-30

**Authors:** Ming Cui, Xian-Shu Gao, Xiaobin Gu, Wei Guo, Xiaoying Li, Mingwei Ma, Shangbin Qin, Xin Qi, Mu Xie, Chuan Peng, Yun Bai

**Affiliations:** ^1^ Department of Radiation Oncology, Peking University First Hospital, Peking University, Beijing, China; ^2^ Graduate School of Medicine, Hebei North University, Zhangjiakou, Hebei, China

**Keywords:** *BRCA2*, mutation, prostate cancer, survival, molecular classification

## Abstract

The aim of this study was to focus on clinicopathological characteristics and prognosis in men with prostate cancer (PCa) harboring a breast cancer 2 (*BRCA2*) gene mutation and to offer convincing evidence to consider BRCA2 mutation as a marker of poor prognosis in the molecular classification of PCa. We searched relevant articles from PubMed, Embase, Web of Science, and the Cochrane Library databases to evaluate the differences in the overall survival (OS) and cancer-specific survival (CSS) between *BRCA2* mutation carriers and non-carriers in patients with PCa. We included 525 *BRCA2* mutation-carriers and 8,463 non-carriers in total from 10 studies in our meta-analysis. The results showed that carrying a *BRCA2* mutation was correlated with a reduced CSS and OS when compared with that of non-carriers, with pooled Hazard Ratios (HRs) of 2.53 (95% confidence interval (CI): 2.10–3.06, *P* < 0.001) and 2.21 (95% CI: 1.64–2.99, *P* < 0.001), respectively. The results also demonstrated that *BRCA2* mutation-carriers harbored a higher Gleason Score (GS) (> 7), TNM stage (> T3, N1, M1), and risk level than non-carriers. Our meta-analysis showed that a *BRCA2* mutation predicted poor survival outcomes in patients with prostate cancer, especially in those undergoing treatments with radiotherapy. Therefore, the use of *BRCA2* mutation as a clinical prognostic factor could help stratify the high-risk patients and provide clinical strategies for more effective targeted treatments for patients with prostate cancer.

## INTRODUCTION

Prostate cancer (PCa) was the most prevalent cancer among males in the United States in 2016 [1]. PCa is not only associated with age, prostate-specific antigen (PSA) levels, the Gleason score (GS), and the TNM stage, but also with family history, especially when female relatives have a history of breast and/or ovarian cancer [2].

In recent times, the treatment of PCa has been facing a bottleneck effect. Evolution into castration-resistant prostate cancer (CRPC) following hormone therapy is observed in an increasing number of patients. At the same time, conventional chemotherapy does not show promising outcomes in CRPC patients with a germline breast cancer 2 (*BRCA2*) mutation.

Although the *BRCA2* mutation is rare and only occurs in approximately 2% of the population with early-onset PCa [3], it increases the risk of PCa by about six times [4–6]. PCa patients with a *BRCA2* mutation have a higher GS scores and have poorer survival outcomes than non-carriers [7, 8].

PSA concentration in serum has always been used to screen early-onset PCa and is regarded as a powerful predictor for PCa risk stratification and of biochemical recurrence and prognosis. Recently, increasing numbers of scientists suggest that testing for PSA alone may not be enough and recommend screening for a *BRCA2* mutation at the start of PCa treatment.

Currently, patients with prostate cancer undergoing radical treatment survive for longer, with the 10-year and 15-year relative survival rates being 98% and 95%, respectively [1]. For prolonged survival in PCa, molecular classification of *BRCA2* mutations has become imperative. Therefore, the objective of this study was to carry out a meta-analysis to focus on clinicopathological characteristics and prognosis in men with PCa harboring a *BRCA2* mutation. We have been able to offer some convincing evidence for regarding *BRCA2* mutation as a marker of poor prognosis in the molecular classification of PCa through this study.

## RESULTS

### Screening and design of the study

**Figure 1:**
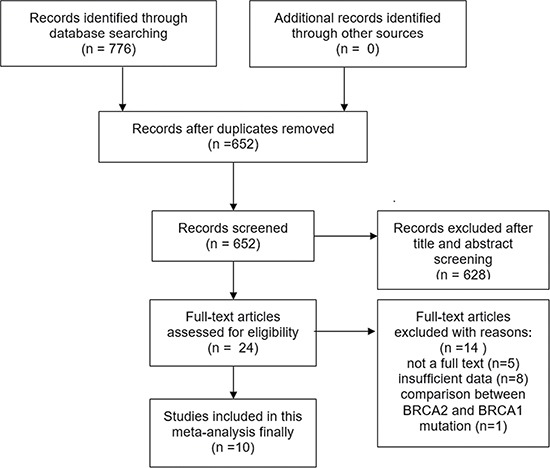
The flow diagram of articles selection

The selection process for our meta-analysis is shown in Figure 1. A search based on keywords on four frequently used databases yielded 776 related articles. The duplicates were eliminated, and 652 articles were screened by reading titles and/or abstracts. After applying the inclusion criteria, only 24 articles remained for full-text screening. Based on selection criteria, 14 articles were excluded for the following reasons: 5 articles because they were meeting abstracts, 8 articles because they contained insufficient data, and 1 article because it had comparisons between *BRCA1* and *BRCA2*. After screening, 10 articles were included in our meta-analysis. All articles were published between 1998 and 2016.

Details of the 10 included studies are shown in Table 1 [8–17]. We included 525 *BRCA2* mutation-carriers and 8,463 non-carriers in total. Nine of the 10 studies were conducted in 6 Western countries including Spain, Australia, Canada, the United States, the United Kingdom, and Iceland. Only 1 study was conducted in an Asian country [9]. The outcomes from 7 studies included only cancer-specific survival (CSS), while the outcome from 1 study included only overall survival (OS). The outcomes from the two remaining studies included both CSS and OS. The Newcastle-Ottawa Scale (NOS) scores for the included studies ranged from 6 to 8, and the results are presented in Table 1.

**Table 1:** Characteristics of included studies

Author	Year	Country	No.of BRCA2 (+)	No.of Non- carriers	Median/Mean age	Follow-up (month)	outcome	Levels of detect BRCA2	Proportion of patients with metastasis	Median/Mean PSA level (ng/ml)	NOS Score
Kim [9]	2016	Korea	212	291	66.0 (44.0–89.0)	44.0 (12–142)	CSS	Protein	3.1%	8.0 (1.0–93.0)	7
Castro [10]	2015	Spain	67	1,235	(+) 58.7 (41.7–77.5) (–) 57.1 (36.0–85.8)	NA	CSS	DNA	Not reported	(+) 8.5 (0.5–68.5)(–) 10.1 (0.5–143)	8
Bolton [11]	2015	Australia	31	59	(+) 65.0 (43.0–84.0) (–) 66.0 (45.0–87.0)	88.8	CSS	Protein	BRCA2 (+): 11.1% BRCA2 (−): 3.3%	(+) 20.95 (0.4–3750) (–) 10.0 (2.0–195)	6
Akbari [12]	2014	Canada	26	1,878	(+) 67.0 (49–90) (–) 65.0	104.4(1.2–144)	CSS	DNA	BRCA2 (+): 25%	(+) 56.3 (–) 13.3	7
Castro [8]	2013	Spain	61	1,940	(+) 57.6 (41.7–88) (–) 57.2 (32.3–88.9)	50.0 (3.5–245)	OS CSS	DNA	Approximately: BRCA2 (+): 31.1% BRCA2 (−): 12.8%	(+) 15.1 (0.5–761) (–) 11.3 (0.2–7800)	8
Thorne [13]	2011	Australia	40	97	(+) 64.9 (43.0–84.0) (–) 66.8 (33.0–87.0)	NA	OS CSS	DNA	BRCA2 (+): 17.5% BRCA2 (−): 4.1%	NA	6
Edwards [14]	2010	UK	21	1,587	NA	NA	OS	DNA	29.4%	NA	6
Gallagher [15]	2010	USA	20	806	(+) 62.0 (40.8–83.0) (–) 68.2 (42.7–94.4)	96	CSS	DNA	Not reported	(+) 7.0 (6.0–9.0) (–) 7.0 (2.0–10.0)	7
Tryggvadóttir [16]	2007	Iceland	30	497	(+) 69.0 (48.0–84.0) (–) 74.0 (50.0–93.0)	NA	CSS	DNA	Approximately: BRCA2 (+): 55.2% BRCA2 (−): 24.6%	NA	7
Edwards [17]	1998	UK	17	73	NA	NA	CSS	DNA	Not reported	NA	6

Abbreviations: NA: not available; CSS: cancer-specific survival; OS: overall survival; PSA: prostate-specific antigen.

### Prognostic value of *BRCA2* mutation on PCa

**Figure 2:**
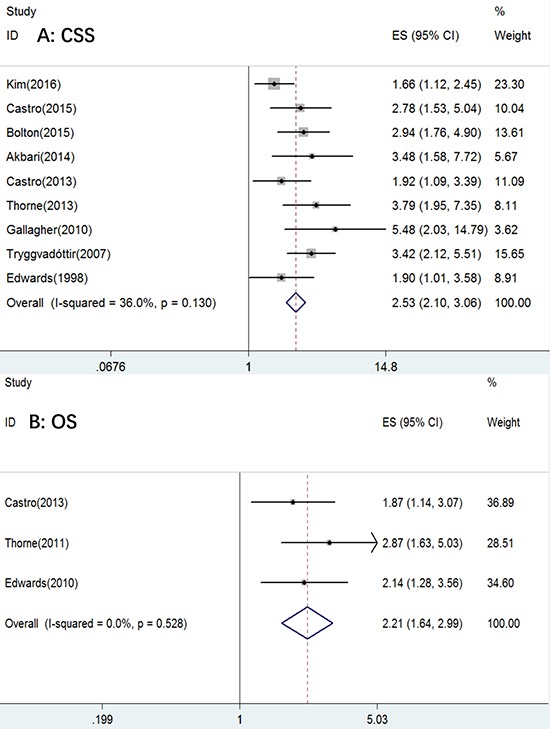
The forest plot of (**A**) CSS and (**B**) OS with BRCA2 mutation.

We included ten studies in total in our meta-analysis for elucidating the effects of *BRCA2* mutation on CSS and OS in patients with PCa. The results are shown in Figure 2 and Table 2. The data indicated that *BRCA2* mutation-carriers exhibited lower CSS and OS than non-carriers, with a pooled HR of 2.53 (95% CI: 2.10–3.06) and 2.21 (95% CI: 1.64–2.99; *P* < 0.01), respectively.

**Table 2:** Main results of the meta-analysis

	Factors	No.of studies	No.of BRCA2(+)	No.of Non-carriers	Effects model	HR (95% cI)	*P*	Heterogeneity	
								I^2^(%)	*P*_h_
CSS	Overall	9	504	6,876	Fixed	2.53 (2.10–3.06)	< 0.001	36	0.13
	Ethnicity								
	Caucasian	8	292	6,585	Fixed	2.88 (2.32–3.58)	< 0.001	0	0.469
	Asian	1	212	291	Fixed	1.66 (1.12–2.45)	0.011	NA	
	Detect methods								
	Protein	2	243	350	Fixed	2.05 (1.50–2.79)	< 0.001	67.1	0.081
	DNA	7	261	6526	Random	2.87 (2.26–3.64)	< 0.001	9.4	0.357
	Sample size								
	< 600	5	330	1,017	Random	2.45 (1.95–3.07)	< 0.001	52.8	0.076
	> 600	4	174	5,859	Fixed	2.75(1.95–3.87)	< 0.001	19.6	0.292
OS	Overall	3	122	3,624	Fixed	2.21 (1.64–2.99)	< 0.001	0	0.528

Abbreviations: P_h_: *P* value of *Q* test for heterogeneity.

No statistically significantly difference heterogeneity was found in CSS among the studies (I^2^ = 36%, P_h_ = 0.13); thus, we performed a subgroup analysis so that we could find the reasons for the heterogeneity. No heterogeneity was found in OS across the studies (I^2^ = 0%, P_h_ = 0.528).

### Subgroup analysis of the effect of *BRCA2* mutations on PCa CSS

To determine the reasons for the heterogeneity seen in CSS and to eliminate the heterogeneity and attain homogeneity among the different subpopulations of patients, we performed a subgroup analysis to investigate the potential sources of heterogeneity across the nine included studies.

The 9 studies that included CSS were divided into 3 subgroups according to 3 different factors: ethnicity, detection methods, and sample size (Table 2). With regard to ethnicity, 8 studies were conducted on Caucasians and 1 study on Asians [9]. *BRCA2* mutation-carriers exhibited lower CSS than non-carriers for both Caucasians and Asians, regardless of the ethnicity, with pooled HRs of 2.88 (95% CI: 2.32–3.58, *P* < 0.001) and 1.66 (95% CI: 1.12–2.45, *P* = 0.011), respectively. The heterogeneity in this case could be eliminated with I^2^ = 0 (P_h_ = 0.13). There were two main methods to detect *BRCA2* mutations at different levels, so we performed a subgroup analysis on detection methods. From among the 9 included studies, 7 studies detected *BRCA2* mutations by extracting DNA from peripheral blood, while 2 studies detected *BRCA2* mutations at the protein level using immunohistochemistry. The results from all the studies showed that *BRCA2* mutation-carriers exhibited lower CSS than non-carriers, regardless of the detection methods used, with pooled HRs of 2.05 (95% CI: 1.50–2.79, *P* < 0.001) in the protein detection subgroup and 2.87 (95% CI: 2.26–3.64, *P* < 0.001) in the DNA detection subgroup. For sample sizes, we selected *n* = 600 as the cut-off value; there were 5 studies with less than 600 cases and 4 studies with more than 600 cases. *BRCA2* mutation-carriers exhibited lower CSS compared with non-carriers even in this subgroup analysis for both the *n* < 600 and *n* > 600 groups, with pooled HRs of 2.45 (95% CI: 1.95–3.07, *P* < 0.001) and 2.75 (95% CI: 1.95–3.87, *P* < 0.001), respectively.

### Association of clinicopathological variables between *BRCA2* mutation-carriers and non-carriers

**Figure 3:**
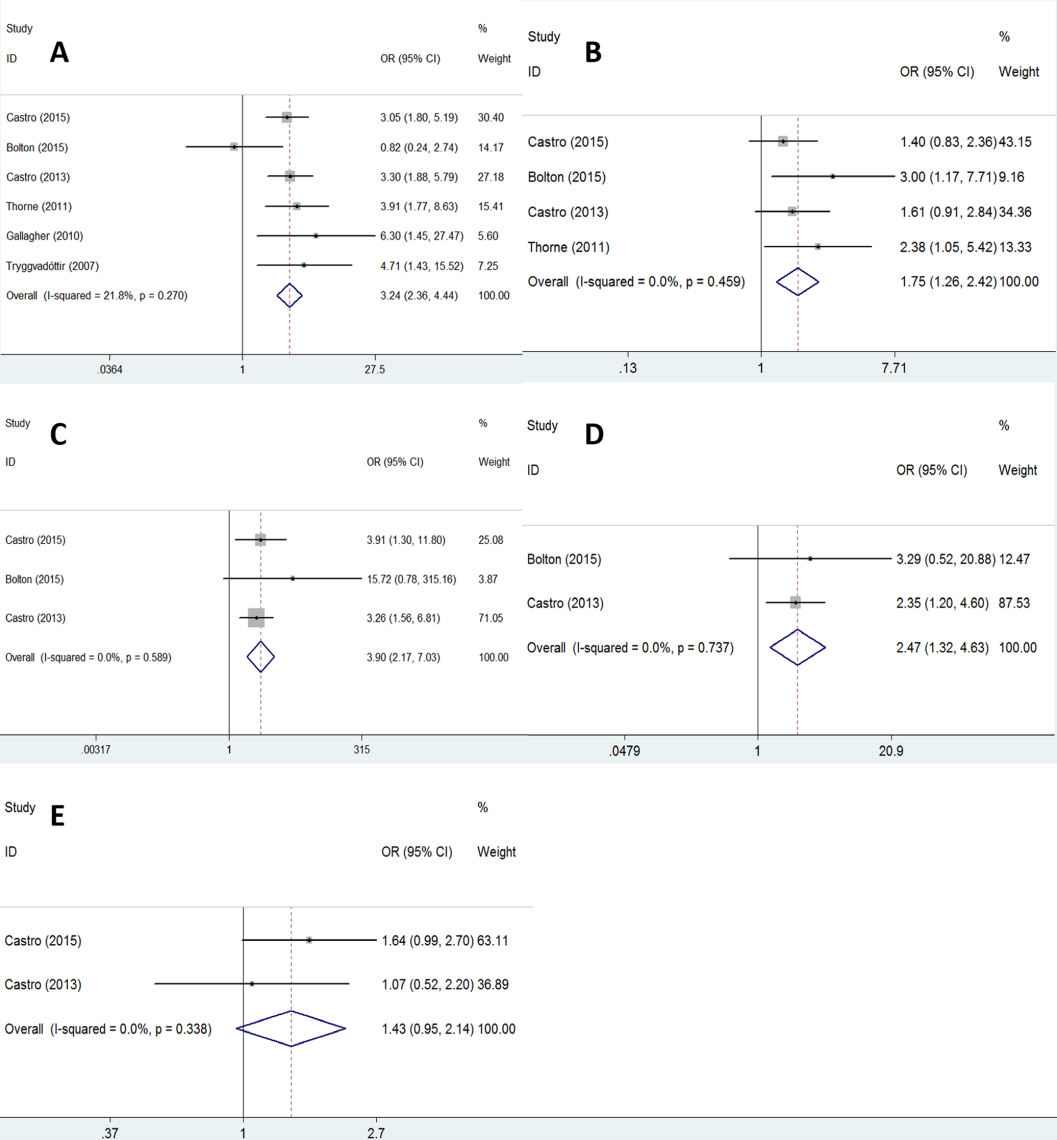
The forest plot of association between clinicopathologic variables and BRCA2 mutation: GS (**A**); T stage (**B**); N stage (**C**); M stage (**D**); risk-stratification (**E**).

To further explore the correlation of clinicopathological variables between *BRCA2* mutation-carriers and non-carriers with PCa, we extracted some impact factors including: GS, TNM stage, and risk stratification of PCa. The results are shown in Table 3 and Figure 3. All the impact factors were associated with a *BRCA2* mutation. Pooled odd ratios (ORs) were GS (> 7 vs. < = 7) (OR = 3.24, 95% CI: 2.36–4.44, *P* < 0.001; Figure 3A), T stage (> = T3 vs. < T3) (OR = 1.75, 95% CI: 1.26–2.42, *P* = 0.001; Figure 3B), N stage (N1 vs. N0) (OR = 3.90, 95% CI: 2.17–7.03, *P* < 0.001; Figure 3C), M stage (M1 vs. M0) (OR = 2.47, 95% CI: 1.32–4.63, *P* = 0.005; Figure 3D), and risk stratification of PCa (high vs. low or intermediate; Figure 3E) (OR = 1.43, 95% CI: 0.95–2.14, *P* = 0.087), respectively.

**Table 3:** Meta-analysis of the association on clinicopathologic features between BRCA2+ and non-carriers with prostate cancer

Variable	No.of studies	No.of BRCA2(+)	No.of Non-carriers	Effects model	OR (95% CI)	*P*	Heterogeneity		Publication begg's p
							I^2^(%)	P_h_	
GS (> 7 vs. < = 7)	6	231	3,722	Fixed	3.24 (2.36–4.44)	< 0.001	21.8	0.27	0.26
T stage (> = T3 vs. < T3)	4	176	2,859	Fixed	1.75 (1.26–2.42)	0.001	0	0.459	0.089
N stage (N1 vs. N0)	3	139	2,367	Fixed	3.90 (2.17–7.03)	< 0.001	0	0.589	0.296
M stage (M1 vs. M0)	2	90	1,999	Fixed	2.47 (1.32–4.63)	0.005	0	0.737	1
Risk (High vs. < High)	2	101	2,510	Fixed	1.43 (0.95–2.14)	0.087	0	0.338	1

Abbreviations: P_h_: *P* value of *Q* test for heterogeneity.

### Sensitivity analysis

**Figure 4:**
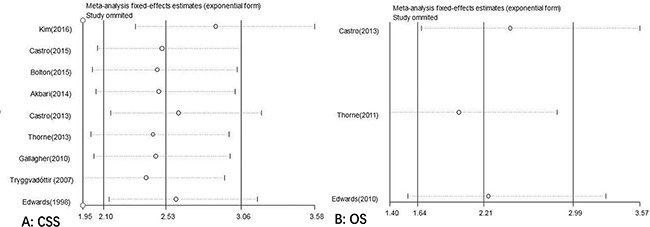
Sensitivities analysis of (**A**) CSS and (**B**) OS with BRCA2 mutation.

To test the results of this meta-analysis, each study was individually eliminated by turn and the pooled data was recalculated. The results of the sensitivity analysis for CSS and OS are shown in Figure 4. Since the corresponding pooled HR did not substantially change, we could confirm our results [18].

### Publication bias

**Figure 5:**
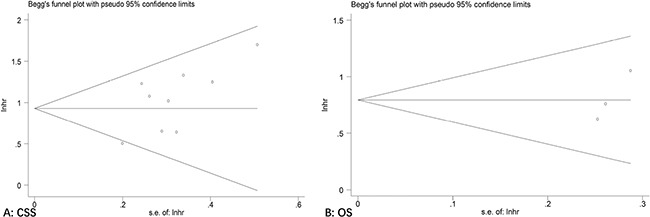
Begg's funnel plot of publication bias test for (**A**) CSS and (**B**) OS with BRCA2 mutation.

Publication bias was assessed using the Begg's and Egger's tests. The results of the Begg's test demonstrated that there was no significant publication bias in CSS (*P* = 0.175) and OS (*P* = 0.296) (Figure 5). The same results could be concluded by using Egger's test for CSS (*P* = 0.07) and OS (*P* = 0.057). Furthermore, the same conclusions were obtained when tested for the correlation of clinicopathological variables with *BRCA2* mutations in PCa (Table 3).

## DISCUSSION

To the best of our knowledge, this is the first meta-analysis that concentrated on PCa screening and prognosis with in patients with *BRCA2* mutations. In this meta-analysis, we demonstrated that a *BRCA2* mutation predicted a lower survival in patients with PCa, both for Caucasians and Asian. Our results were consistent with those of other studies, which show that a *BRCA2* mutation not only enhances the risk of PCa, but also doubles the PSA levels [8, 19, 20]. For this reason, only PSA screening may not be enough for patients with PCa at early stages, so we suggest that the *BRCA2* gene be screened routinely for mutations as a biomarker of poor prognosis in the molecular classification of PCa, thus offering scope in the planning for more effective clinical strategies for treatment.

A *BRCA2* mutation was first detected in breast or ovarian cancer in females and was significantly associated with family history, especially when relatives had breast, ovarian, or prostate cancer. First, family history increases the risk of morbidity associated with PCa by about twice than that seen for patients in the general population and increases the risk of early onset (diagnosed before 60 years of age) by about four times when compared with the general population. This is a direct influence of family history on PCa onset and prognosis. Second, it is thought that families with a clearly dominant predisposition to breast or ovarian cancer harbor germline mutations in *BRCA2* genes. *BRCA2* mutations predicted poor survival outcomes as seen in our meta-analysis. *BRCA2* mutations are known to elevate the risk of not only breast and ovarian cancer, but also other cancers like PCa. This is an indirect influence of family history on PCa onset and prognosis [2–5].

*BRCA2* mutations were also associated with GS, TNM stage, and risk stratification, as demonstrated by our meta-analysis (Table 3). With regard to the GS, our results indicated that GS > 7 was more frequent in *BRCA2* mutation-carriers than non-carriers. Mitra et al. also observed that the GS was higher in *BRCA2* mutation-carriers [7]. A higher GS correlates with poor survival and a higher requirement for needle biopsy, particularly in patients with GS 8–10 [21]. With regard to the TNM stage, our results showed that *BRCA2* mutations were correlated with T3–T4, N1, and M1 stages. A high TNM stage also correlates with high mortality in PCa and is a predictor of poor prognosis, which was also found in other studies [8, 9]. PCa prognosis also depends on risk stratification; each risk level has corresponding treatments in the National Comprehensive Cancer Network (NCCN) guidelines. This meta-analysis showed that risk stratification was also relevant to *BRCA2* mutations. *BRCA2* mutation-carriers had a higher risk than non-carriers, though there was no statistically significantly difference (*P* = 0.087) due to the limited number of studies (only two studies) included in this group.

We analyzed the proportion of PCa patients with metastasis (N1 or M1) in all included studies and found that *BRCA2* mutation-carriers have a significantly higher rate of metastasis than non-carriers. This implies that *BRCA2* mutation-carriers had a poorer prognosis with a lower metastasis-free survival (MFS) after treatment than that seen in non-carriers.

*BRCA2* mutations were also correlated with radiotherapy outcomes. In Castro et al. [13], a *BRCA2* mutation predicted poor CSS in all patients. However, in the subgroup analysis, no significant differences after radical prostatectomy (RP) were observed between *BRCA2* mutation-carriers and non-carriers (*P* = 0.566). However, CSS after radiation therapy (RT) was significantly higher in non-carriers than in mutation-carriers (*P* < 0.001). This indicated that a *BRCA2* mutation may predict poor CSS after RT rather than RP, but this conclusion needs many clinical trials for verification.

After exposure to ionizing radiation in human cells, DNA lesions can be observed due to DNA single-strand breaks (SSBs) and double-strand breaks (DSBs [22]. When DNA strands break, repair mechanisms are initiated. SSBs are mostly repaired by base excision repair (BER), nucleotide excision repair (NER), or mismatch repair pathways using an intact complementary DNA strand as a template. DSBs are the foremost form among all the lesions and its repair plays an important role in maintaining genomic integrity and stability. The repair pathways of DSBs in human cells are non-homologous end-joining (NHEJ) and homologous recombination (HR) [23–26]. BRCA2 is considered to enhance genomic stability through error-free repair of DSBs by HR [27, 28]. A *BRCA2* mutation makes this unavailable and the repair pathways transition from HR to NHEJ, leading to genomic instability [28, 29].

Poly (ADP-ribose) polymerase-1 (PARP1) had a long exploring history since 1963 [30]. PARP1 is associated with the repair of SSBs through BER [31, 32]. When PARP1 function is inhibited, DNA lesions may be repaired by HR instead of BER. Under this circumstance, if *BRCA2* is also mutated, the dual effects make repair abnormal and lead to cancer cell death [28]. According to this theory, PARP1 inhibition could be used in *BRCA2* mutation-carriers to obtain satisfactory therapeutic outcomes. Some studies demonstrated that PARP1 inhibition was highly selective in human tumors with *BRCA2* mutations [33]. Olaparib, an inhibitor of PARP1, obtained accelerated approval from the Food and Drug Administration (FDA) in December 2014. It had shown promising therapeutic effects in *BRCA2* mutation-carrier patients with triple-negative breast cancer and sporadic serous ovarian cancer in females and CRPC and metastatic PCa in males [32–34].

Patients with metastatic PCa and CRPC are thought to be associated with DNA repair defects like those seen with *BRCA2* mutations. These patients can benefit from Olaparib than *BRCA2* mutation non-carriers with prolonged OS (median, 9.8 vs. 2.7 months; *P* < 0.001) and PFS (median, 13.8 vs. 7.5 months; *P* = 0.05) [34].

There are two major strengths of this meta-analysis. First, we have an enormous sample size with 8,988 patients (525 *BRCA2* mutation-carriers and 8,463 non-carriers), which make the results convincing. Second, both Caucasian and Asian patients are included in this meta-analysis; therefore, our results are applicable in these two ethnic populations.

There are three limitations in this meta-analysis. First, our results are concluded from 10 articles that are all retrospective studies, which provide less sufficient clinical evidence than randomized control trial (RCT) studies. Second, only one study involve Asian men and may be inadequately representative. Finally, the higher incidence of PCa in African-Americans demonstrates that genetic factors are an important determinant of the variations in risk at the population level [2]. However, our included studies do not contain this population, and therefore, a subset analysis could not be performed for this ethnicity.

In conclusion, our meta-analysis showed that patients with PCa harboring a *BRCA2* mutation had poor survival both in Caucasian and Asian populations. Therefore, a *BRCA2* mutation as a clinical prognostic factor could help stratify the high-risk patients and provide inputs in the planning of specific and more effective treatments. Thus, we suggest that *BRCA2* mutation as a biomarker of poor prognosis could contribute in the molecular classification of PCa.

## MATERIALS AND METHODS

This meta-analysis was performed following the Preferred Reporting Items for Systematic Reviews and Meta-Analyses (PRISMA) Statement guidelines strictly [35].

### Literature search

We searched relevant articles that were published prior to July 31, 2016 from PubMed, Embase, Web of Science, and the Cochrane Library databases. The key terms included “Breast Cancer 2” or “BRCA2,” “genes,” “mutation,” “survival,” “prognosis” or “prognostic,” “predict” or “predictive,” “treatment,” “biomarker,” and “prostate cancer” or “prostate neoplasms” or “prostate carcinoma” or “PCa.” The searching strategies were keywords combined with a manual search from references in all eligible studies.

### Inclusion and exclusion criteria

Articles were selected on the basis of the following inclusion criteria: (1) comparison between PCa patients with a *BRCA2* mutation and non-carriers; (2) a *BRCA2* mutation was confirmed in postoperative pathological specimens or detected in the plasma; (3) survival-associated outcomes including OS, CSS, MFS, progression-free survival (PFS), disease-specific survival (DSS) and biochemical recurrence-free survival (bRFS); (4) sufficient data extraction from the studies to enable calculation of HRs and 95% CIs; and (5) the sample sizes larger than 30.

Furthermore, the studies were excluded if: (1) studies were not performed on human subjects but on cells or animals; (2) articles were not written in English; (3) the data were insufficient for calculation; or (4) articles were not full-text or were review articles.

### Data extraction and quality assessment

Two researchers (M.C and XB.G) extracted the data from the eligible studies independently, and any disagreements were resolved by reconsidering and discussing with our senior investigator (XS.G). The following data were extracted from the eligible studies according to a predefined spreadsheet: first author's name, year of publication, author's country, number of patients with a *BRCA2* mutation, number of non-carriers, age, follow-up duration, survival outcomes, levels of detected BRCA2, proportion of patients with metastasis, PSA levels, and NOS scores.

Quality assessment was performed on each study by three investigators independently (M.C, W.G, and XB.G) using the NOS quality assessment criteria [36]. The NOS scoring system uses stars to evaluate the methodological quality on three aspects: selection, comparability, and outcome. Each aspect has two to four stars out of a total of nine stars for the NOS scoring system. We regarded studies with more than six stars as high-quality studies.

### Statistical analysis

The STATA 12.0 software (Stata Corp LP, College Station, TX, USA) was used to perform our meta-analysis. HRs, ORs, and their 95% CIs were calculated from the included studies. HR > 1 meant lower survival rates for BRCA2-mutation carriers compared with non-carriers [37]. We extracted HRs and 95% CIs from every eligible study and for those studies from which the data could not be extracted directly, we estimated the date from survival curves [38].

### Assessment of heterogeneity

The heterogeneity of pooled results was tested with the Cochran's *Q* test and Higgins I-squared statistic. We defined significant heterogeneity if I^2^ > 50% and P_h_ value < 0.1 at the same time. Under this circumstance, the random effects model would be chosen. Otherwise, the fixed effects model was selected.

### Assessment of reporting bias

Publication bias was evaluated by the Begg's and Egger's funnel plot tests, and *P* < 0.05 was thought to be statistically significant. For evaluating the stability of the results, we performed a sensitivity analysis.

### Subgroup analysis

For subgroup analysis, all studies were divided into subgroups based on ethnicity detection methods and sample size. Association analysis was also performed for variables including GS, TNM stage, and risk stratification of PCa.

## Abbreviations

PCa: Prostate cancer
BRCA2: Breast Cancer 2
OS: Overall survival
CSS: Cancer-specific survival
HRs: Hazard Ratios
CI: Confidence interval
ORs: Odds ratio s
PSA: Prostate-specific antigen
GS: Gleason score
CRPC: Castration-Resistant Prostate Cancer
NOS: Newcastle-Ottawa Scale
SSB: Single-strand breaks
DSB: Double-strand breaks
BER: Base excision repai r
NER: Nucleotide excision repair
NHEJ: Non-homologous end-joining
HR: Homologous recombination
PARP-1: Poly (ADP-ribose) polymerase-1
FDA: Food and Drug Administration
RCT: Randomized control trial
PRISMA: Preferred Reporting Items for Systematic Reviews and Meta-Analyses
PFS: Progression-free survival
DSS: Disease-specific survival
MFS: Metastasis-free survival
bRFS: biochemical recurrence free survival
NCCN: National Comprehensive Cancer Network
